# Clinically significant tumor histology in suspected primary bladder cancer: is every transurethral resection necessary?

**DOI:** 10.3389/fsurg.2026.1813017

**Published:** 2026-06-12

**Authors:** Conrad Leitsmann, Alexander Stephan Reese, Richard Zigeuner, Hanna Zurl, Klara Pohl, Johannes Mischinger, Iva Simunovic, Carl Ketterer, Florestan Koll, Sebastian Mannweiler, Marianne Leitsmann, Sascha Ahyai

**Affiliations:** 1 Department of Urology, Medical University of Graz, Graz, Austria; 2 Center for Surgery and Public Health, Brigham and Women’s Hospital, Harvard Medical School, Boston, MA, United States; 3 Department of Pathology, Medical University of Graz, Graz, Austria; 4 aQua-Institute for Applied Quality Improvement and Research in Health Care, Goettingen, Germany

**Keywords:** bladder cancer, diagnostic setup, malignancy, overtreatment, predictive parameters, TURBT

## Abstract

**Purpose:**

Transurethral resection of bladder tumor (TURBT) is the standard for diagnosis and initial treatment of non-muscle-invasive bladder cancer (NMIBC) a disease with highly variable presentation. To evaluate the rate of malignancy in patients undergoing elective TURBT for suspected BC.

**Methods:**

A retrospective analysis of 217 patients undergoing primary TURBT for BC suspicion at our tertiary care center between January and June 2023 was performed.

Key parameters included preoperative macroscopic cystoscopy findings, intraoperative macroscopic findings, and pathologic tumor characteristics from the TURBT specimen. The primary outcome was the detection rate of malignancy. Secondary outcomes included the correlation of the interobserver congruence on preoperative and TURBT findings. Multivariate logistic regression was performed to identify significant predictors of malignancy.

**Results:**

Of 217 patients, 35% had no malignancy in the TURBT specimen. Interobserver congruence was observed in 80.6% of cases. Papillary findings in the initial cystoscopy (Odds ratio (OR) = 5.6, 95% Confidence interval (CI) = 2.18–14.52, *p* < 0.001), age (OR = 1.057, 95%CI = 1.02–1.1 *p* = 0.002), interobserver congruence on preoperative and TURBT findings (*p* < 0.001, OR = 99.3, 95%CI = 10.9–903.9), and tumor size (OR = 1.9, 95%CI = 1.33–2.71, *p* < 0.001) were significant predictors for malignancy.

**Conclusion:**

Direct TURBT may not be obligatory for all patients with suspicious bladder lesions. Our findings highlight the importance of a thorough preoperative diagnostic setup to avoid unnecessary procedures and spare the burden for patients. There is a need to establish more selective criteria for TURBT.

## Introduction

Bladder cancer, particularly non-muscle-invasive bladder cancer (NMIBC), is recognized as a highly heterogeneous disease, marked by considerable variability in its risk for recurrence and progression, but also in appearance. Clinical symptoms are not a reliable indicator of malignancy. Among patients presenting with visible hematuria, approximately 20−25% are found to have BC (1). While imaging modalities such as ultrasound can detect some tumors, cystoscopy remains the definitive tool to substantiate the suspicion of BC (2). BC presents with various cystoscopic characteristics, such as papillary formations, red spots, or flat lesions (3). Sequentially, the standard approach for the diagnosis, staging, and initial treatment of NMIBC is elective transurethral resection of bladder tumor (TURBT) (4).

In clinical practice, patients are frequently referred for TURBT due to suspected BC, although histology later reveals benign pathology. At the same time, healthcare systems face increasing strain — amplified by the COVID-19 pandemic and a shortage of trained personnel, particularly nursing staff — resulting in cancelled procedures, closure of operating theatres, and prolonged waiting times for elective urological interventions (5). These challenges, alongside the well-established association between treatment delays and adverse oncological outcomes (6), underscore the need for precise indication management and raise questions regarding the necessity of TURBT in all suspected cases.

Further considering the substantial psychological and physical burden that a potential cancer diagnosis or recurrence imposes on patients, it is imperative to identify factors that may argue against immediate TURBT in selected cases. Avoiding unnecessary procedures in appropriately stratified patients could help alleviate pressure on operating theaters and optimize resource allocation, while also reducing emotional distress, procedural risks, and recovery time.

In this setting, we evaluated the detection rate of malignancy in patients undergoing TURBT and explored potential factors that could help to avoid unnecessary procedures by optimizing the diagnostic set up when BC is suspected.

## Materials and methods

### Study population

We conducted a retrospective review of medical records of patients who underwent TURBT for suspected BC at our tertiary care center, between January and June 2023. All patients were referred for TURBT by a board-certified urologist, either in the outpatient setting or at the hospital. Standard TURBT was performed according to current guidelines by a board-certified surgeon or a urology resident under supervision. The study was approved by the ethics committee of the Medical University Graz (EC number: 36-099 ex 23/24).

### Parameters

Baseline patient characteristics included gender, age, body mass index (BMI), tobacco smoking status, pack-years of smoking, and the American Society of Anesthesiologists (ASA) classification as a proxy for preoperative health status. Symptoms reported by the patient such as macrohematuria were recorded, as well as a history of BC. Preoperative diagnostic parameters captured whether preoperative cystoscopy was performed, the description of papillary findings in the initial cystoscopy, and whether the suspicion of BC was determined by an outpatient or a hospital-based urologist.

The primary outcome was the detection rate of malignancy in the pathological specimen of the performed TURBT. Secondary outcomes included the interobserver congruence on preoperative and TURBT findings. TURBT findings were obtained from the surgical report. Further, type of anesthesia (spinal or general anesthesia), tumor count, tumor size, the use of photodynamic cystoscopy and operation time were recorded for each case. Also, early postoperative instillation therapy was noted. Postoperative complications were categorized according to the Clavien—Dindo classification (7).

Pathological tumor characteristics were assessed using the current WHO Classification of Tumors, 5 th Edition, Urinary and Male Genital Tumors (8) and TNM Classification of Malignant Tumors, Eighth Edition (9), with tumors categorized as pTa (low grade or high grade), pT1, pT2, pT3, and pTis. The presence or absence of muscularis invasion was documented, along with the occurrence of additional carcinoma *in situ* (Cis).

### Statistical analysis

Continuous variables, such as age, tumor size, and operation time, were expressed as mean ± standard deviation (SD) or median median ± interquartile range (IQR). For the comparison of continuous variables, the t-test was used for normally distributed data, while the Mann–Whitney U test was employed for non-normally distributed data. Categorical variables, such as gender, tumor stage, and postoperative complications, were presented as frequencies and proportions, and comparisons were made using the chi-square test.

To illustrate correlations between malignant histology and respective parameters, we divided the patient cohort into two groups: those with malignancy and those without. Univariate analyses were then conducted to assess differences between the two groups based on parameters such as age, tumor count, tumor size, and interobserver congruence on preoperative and TURBT findings.

Multivariable binary logistic regression models were employed to assess predictors for malignancy in TURBT samples. Covariates included in the regression model were patient age, papillary findings in initial cystoscopy, cystoscopic confirmation of preoperative findings by the surgeon during TURBT, tumor count, and tumor size. Statistical analyses were performed using SPSS version 29.0.2 (IBM Corp., Armonk, NY). Significance was set at *p* < 0.05 for all analyses.

## Results

### Baseline characteristics

A total of 217 patients were included in the study, with a mean age of 70.5 ± 12 years. The cohort consisted of 164 males (76%) and 53 females (24%) (Table 1).

**Table 1:** Baseline patient characteristics (*n* = 217).

Parameters	Total cohort (*n* = 217)	No malignancy (*n* = 77)	Malignancy (*n* = 140)	*p*-value
Gender (m, f; *n* (%))	164 (76), 53 (24)	54 (70.1), 23 (29.9)	123 (78), 34 (22)	0.19
Age (mean ± SD)	70.5 (± 12)	67.2 (± 14)	72.3 (± 10.9)	**0** **.** **02**
BMI (mean ± SD)	27.2 (± 5)	27.1 (± 5.7)	27.3 (± 4.5)	0.47
Tobacco smoking, *n* (%)	65 (30)	19 (24.7)	46 (32.9)	0.22
Packyears (mean ± SD)	18.2 (± 29.9)	20 (± 31.6)	15 (± 26.5)	0.51
ASA (%)				0.13
1	13 (6)	8 (10.4)	5 (3.6)	
2	70 (32.3)	24 (31.2)	46 (32.9)	
3	99 (45.6)	37 (48.1)	62 (44.3)	
4	33 (15.2)	8 (10.4)	25 (17.9)	
History of BC (%)	64 (29.4)	26 (33.8)	38 (27.1)	0.31

m, male; f, female; n, number; SD, standard deviation; BMI, body mass index; ASA, American Society of Anesthesiologists; BC, bladder cancer.

Bold values indicate statistical significance.

The average BMI was 27.2 ± 5. Tobacco use was reported in 30% of patients, with an average of 18.2 ± 29.9 pack-years. Regarding preoperative health status, ASA classifications were distributed as follows: 6% of patients were ASA 1, 32.3% ASA 2, 45.6% ASA 3, and 15.2% ASA 4, meaning that 61% of the cohort had an ASA score of 3 or higher. A history of BC was documented in 29.4% of patients.

Macrohematuria was the initial symptom in 45.2%. Urine cytology was taken in 9.7% of the total cohort. Preoperative cystoscopy was performed in 74.2% of patients, and in 30.4% of those no papillary findings were observed. The majority of patients (75.1%) were referred for surgery by an outpatient urologist (Table 2).

**Table 2:** Preoperative parameters of the total cohort (*n* = 217).

Parameters	Total cohort (*n* = 217)	No malignancy (*n* = 77)	Malignancy (*n* = 140)	*p*-value
Macrohematuria as initial symptom (%)	98 (45.2)	25 (32.5)	73 (52.1)	**0** **.** **002**
Preoperative urine cytology (%)	21 (9.7)	8 (10.3)	13 (9.2)	**0**.**83**
Preoperative cystoscopy (%)	161 (74.2)	57 (74.0)	104 (74.3)	0.97
History of history of bladder cancer	64 (29.4)	26 (33.8)	38 (27.1)	0.31
No papillary finding in initial cystoscopy (%)	66 (30.4)	46 (59.7)	20 (14.3)	**<0.001**
Referral to surgery by an outpatient urologist (%)	163 (75.1)	57 (74)	106 (75.7)	0.78

Bold values indicate statistical significance.

75.1% of procedures were performed under general anesthesia, with spinal anesthesia used in 24.9% of cases. The mean tumor count was 1.9 ± 1.7, while the median tumor size measured 1.5 (IQR: 0.3–2.8) cm. The maximum size was 8.5 cm. The median operation time was 27 (IQR: 16−46) minutes. Intraoperative photodynamic techniques were utilized in 5.5% of cases, and preoperative cystoscopic findings were confirmed by the surgeon during TURBT in 80.6% of cases. Early postoperative instillation therapy was administered in 28.2% of patients. According to the Clavien—Dindo classification, 8.3% of patients experienced grade 1 complications, 0.5% had grade 2, and 0.9% had grade 3b complications (Table 3).

**Table 3:** Intra- and postoperative parameters (*n* = 217).

Parameters	Total cohort (*n* = 217)	No malignancy (*n* = 77)	Malignancy (*n* = 140)	*p*-value
Type of anesthesia				**0** **.** **3**
Spinal anesthesia	54 (24.9)	16 (20.8)	38 (27.1)	
General anesthesia	163 (75.1)	61 (79.2)	102 (72.9)	
Tumor count (mean ± SD)	1.9 ± 1.7	1.4 ± 1.4	2.1 ± 1.8	**<0** **.** **001**
Tumor size (cm; median, IQR)	1.5 (0.3–2.8)	0.7 (0–1.4)	2 (1–3)	**<0** **.** **001**
Mean operation time (min, median, IQR)	27 (16–46)	21 (13–30)	33 (18–54)	**<0** **.** **001**
Complications according to Clavien—Dindo (%)				0.24
1	18 (8.3)	3 (3.9)	15 (10.7)	
2	1 (0.5)		1 (0.7)	
3b	2 (0.9)	0 (0)	2 (1.4)	
Early postoperative installation therapy (%)	62 (28.2)	6 (7.8)	56 (40)	**<0** **.** **001**
Interobserver congruence on preoperative and TURBT findings (%)	175 (80.6)	36 (46.8)	139 (99.3)	**<0** **.** **001**
Intraoperative photo dynamic technique (%)	12 (5.5)	10 (13)	2 (1.4)	**<0** **.** **001**

Bold values indicate statistical significance.

**Figure 1:**
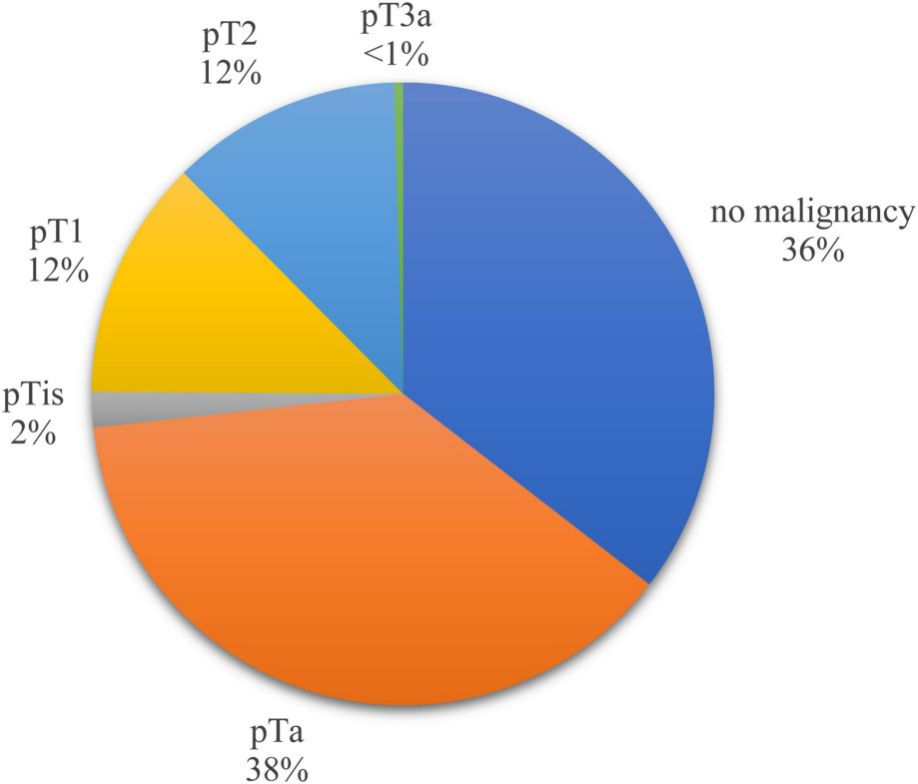
Histopathological findings (*n* = 217).

Histopathological examination revealed that 35% of patients (*n* = 77) had no malignancy. Among the 140 patients diagnosed with BC, tumor classification according to the WHO system was as follows: 18.6% were classified as pTa low grade, 40% as pTa high grade, 19.3% as pT1 (all high grade), 18.6% as pT2, 0.7% as pT3, and 2.9% as pTis (Figure 1). Additionally, Cis was present in 14.3% of cases.

### Predictors of malignancy

**Figure 2:**
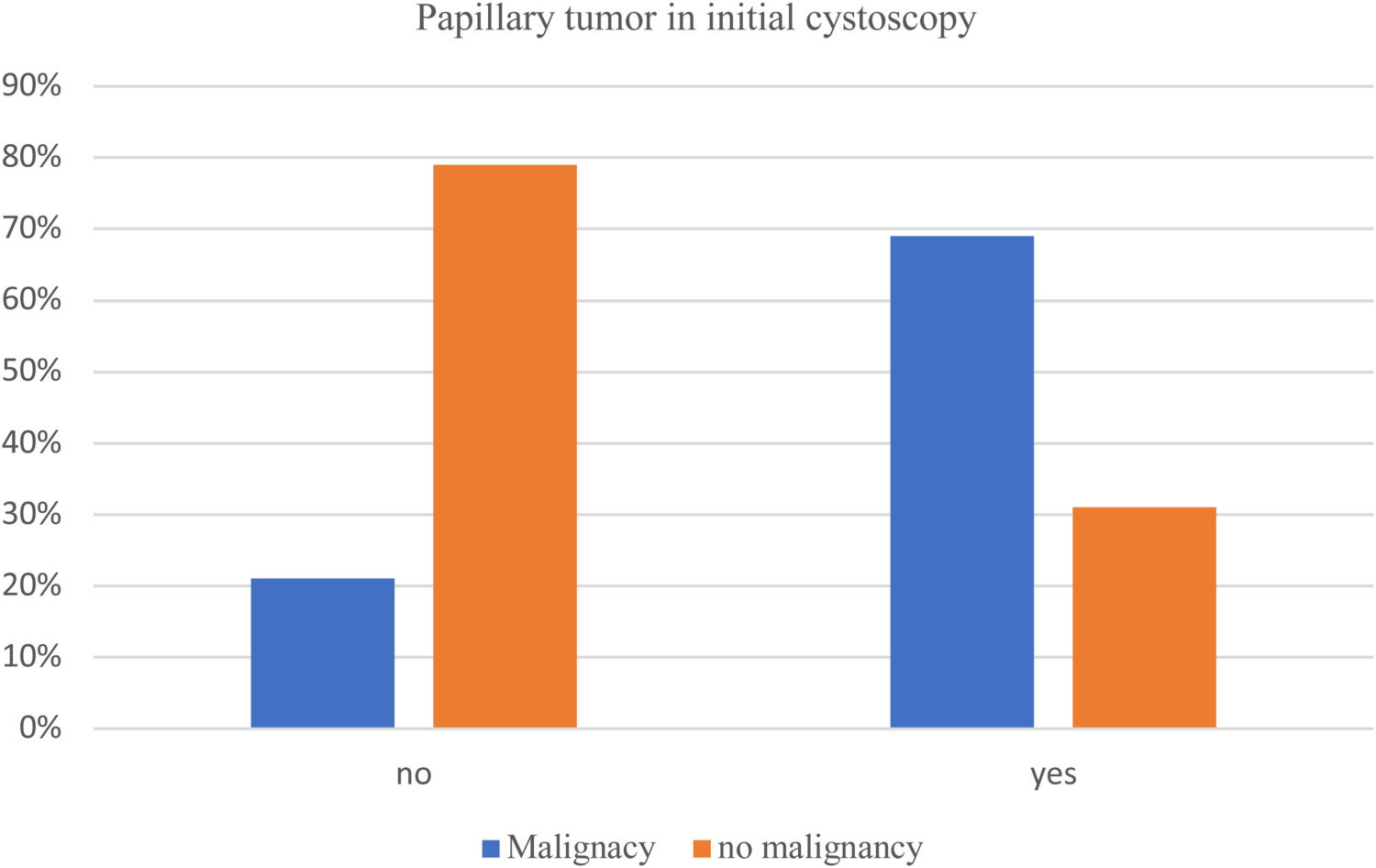
Intraoperative correlation with papillary tumor in initial cystoscopy (*n* = 217, *p* < 0.001).

Papillary findings strongly correlated with malignancy (*p* < 0.001, Figure 2).

Macrohematuria was observed at a significantly higher rate in the malignancy group (52.1%) compared to the non-malignancy group (32.5%, *p* = 0.002). However, there was no significant difference in the use of preoperative urine cytology between patients with malignancy (10.3%) and those without (9.2%, *p* = 0.83, Table 2).

Patients in the malignancy group had significantly more tumors (2.1 ± 1.8) compared to the non-malignancy group (1.4 ± 1.4, *p* < 0.001). Additionally, tumors were larger in the malignancy group (2 (IQR: 1-3) cm vs. non-malignancy group: 0.7 (IQR: 0-1.4) cm, *p* < 0.001). Procedure duration was also significantly longer in the malignancy group (46.7 ± 49.8 min) compared to the non-malignancy group (27.7 ± 31 min, *p* < 0.001, Table 3).

**Figure 3:**
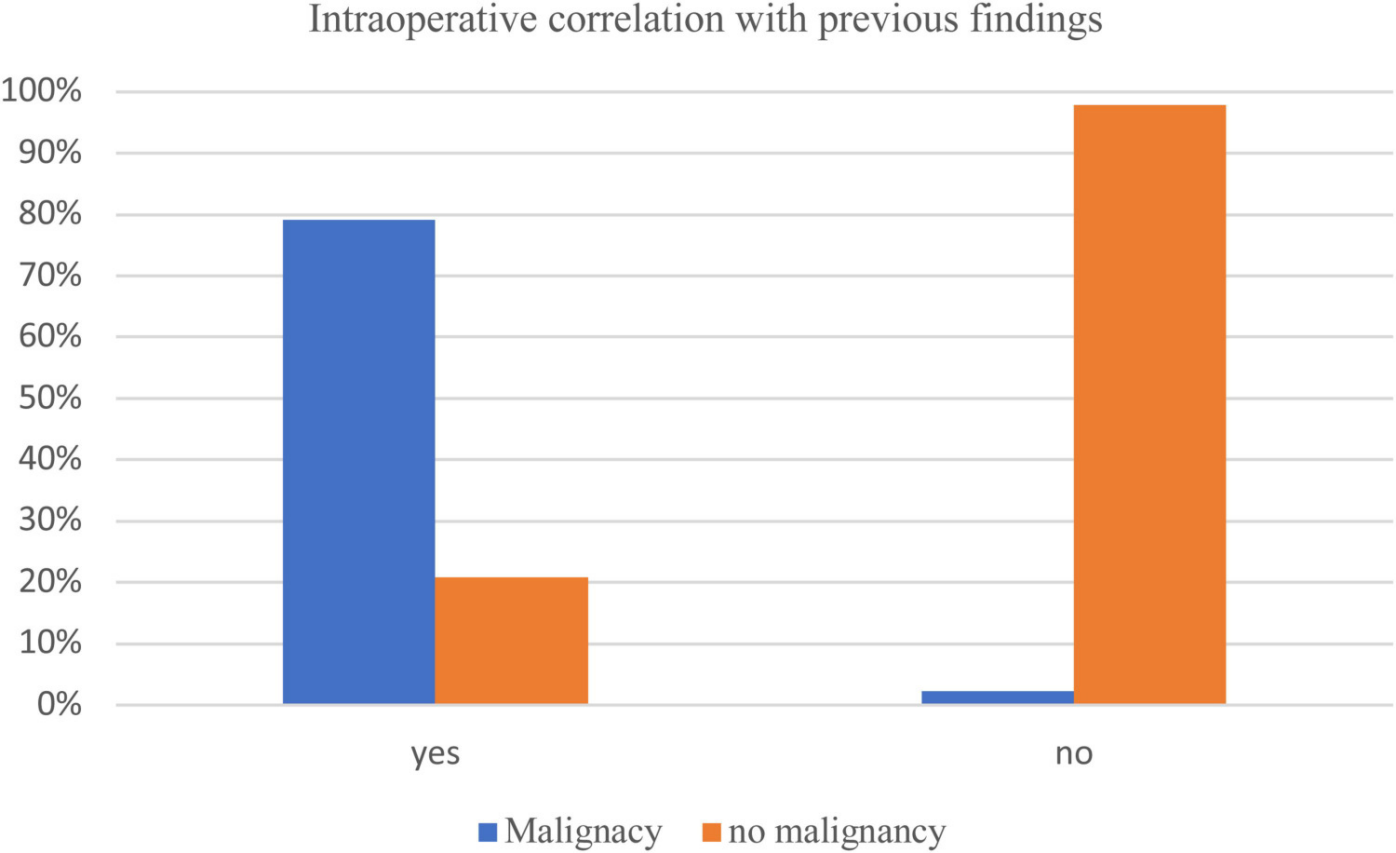
Interobserver congruence on preoperative findings during TURBT (*n* = 217, *p* < 0.001).

The interobserver congruence on preoperative findings during TURBT showed a strong statistical correlation with malignancy. Among patients without malignancy, the confirmation rate was significantly lower (46.8%), compared to 99.3% in patients with malignancy (*p* < 0.001). Notably, in cases where there was no concordance between preoperative and intraoperative findings, 98% of patients did not have malignancy (Figure 3).

In the multivariable analysis, age (OR = 1.057, 95%CI = 1.02-1.1, *p* = 0.002), papillary findings in the initial cystoscopy (OR = 5.6, 95%CI = 2.18-14.52, *p* < 0.001), and cystoscopic confirmation of preoperative findings during TURBT (OR = 99.3, 95%CI = 10.9-90.3, *p* < 0.001) were significant predictors for malignancy in the TURBT specimen. Tumor size also showed a significant association with malignancy (OR = 1.9, 95%CI = 1.33-2.71, *p* < 0.001; Table 4).

**Table 4:** Multivariable analysis of predictors for malignancy in the TURBT sample (*n* = 217).

Parameters	*p*-value	Odds ratio	95%Confidence interval
Age	0.002	1.057	1.02–1.1
Papillary finding in initial cystoscopy	**<0** **.** **001**	5.6	2.18–14.52
Tumor count	0.297	1.13	0.9–1.4
Intraoperative correlation with previous finding	**<0** **.** **001**	99.3	10.9–903.9
Tumor size	**<0** **.** **001**	1.9	1.33–2.7

Bold values indicate statistical significance.

## Discussion

Our study assessed the malignancy rate in patients undergoing elective TURBT for suspected BC, revealing that 35% had no malignancy in the resected specimen. In a similar study setting5 including 81 patients, the proportion of benign histology was 22% (10).

Moreover, we found that only 80.6% of initial macroscopic cystoscopic findings were confirmed intraoperatively. The similar rates of confirmation among in-house and outpatient urologists at least suggest a consistent diagnostic accuracy across different clinical settings. Mitropoulos et al. compared the assessment of urothelial lesions during cystoscopy by specialists with the histological results and found that in 89.7% (131 of 146 of cases), the diagnosis was correct (11). Dekalo et al. focused on the precise description of tumor stage and grade of urothelial lesions during cystoscopy. The researchers concluded that while urologists correctly determined stage and grade in 63.5% of cases, diagnostic accuracy improved significantly when multiple experienced urologists analyzed the same images, reaching a majority consensus (12). Interobserver congruence on preoperative and TURBT findings emerged as a strong predictor of malignancy. Nevertheless, the remaining 19.4% of inconsistent cases detected in our study underline the need to improve diagnostic precision.

Other key factors associated with malignancy were papillary findings in the initial cystoscopy and tumor size. This further underscores the value of thorough cystoscopic assessment to optimize patient assessment, specifically the need for accurate tumor evaluation with detailed documentation of all macroscopic features. As strongly recommended by the European Association of Urology (EAU) guidelines it is essential to record characteristics such as site, size, number, and appearance, as well as any mucosal abnormalities. The use of a bladder diagram to visualize and document these features can aid in precise diagnosis and follow-up (3). This level of detailed documentation can improve intraoperative accuracy and contribute to better treatment planning and patient outcomes, especially in cases where preoperative findings may not correlate with intraoperative observations (13).

Additionally, guidelines strongly recommend the use of voided urine cytology as an adjunct to cystoscopy for the detection of high-grade tumors. to improve diagnostic accuracy. In our study, urine cytology was only taken in 10% of patients, indicating room for improvement (3). However, considering the low sensitivity of cytology for detecting low-grade tumors, there has been growing interest in developing urinary biomarkers for the early detection of BC. Laukhtina et al. conducted a systematic review demonstrating that certain biomarkers achieved sensitivity levels of up to 93%, specificity of up to 84%, with a positive predictive value of 67% and a negative predictive value of 99% (14). Despite these promising findings, no biomarker has yet demonstrated sufficient clinical utility to be integrated into routine practice, and none has been incorporated into national or international guidelines to date (3).

Cystoscopy remains the gold standard for BC diagnosis. Recent advancements, including the use of photodynamic cystoscopy (PDD) and narrow band imaging (NBI) have demonstrated improved diagnostic sensitivity over conventional techniques (15, 16). The EAU guidelines recommend fluorescence-guided biopsy and resection, highlighting their superior sensitivity in detecting malignant tumors, especially Cis (3). We used photodynamic diagnosis (PDD) in selected cases, particularly in patients who were referred to us with less clearly papillary findings. The integration of these technologies has the potential to enhance patient outcomes and reduce the likelihood of missed diagnoses, especially given that PDD was utilized in only 5.5% of our cohort and none of the other techniques. It is also feasible to consider their use in outpatient settings, eliminating the need for inpatient admission to a urology department.

In addition, new technologies such as augmented cystoscopy with deep learning promise improved BC detection. A recent pilot study demonstrated the feasibility of using artificial intelligence (AI)-assisted cystoscopy (CystoNet) during cystoscopy and TURBT to provide active feedback to surgeons. This technology has the potential to optimize real-time cystoscopy dynamics, that enhances detection rates and may further reduce unnecessary procedures (16).

Developments in BC imaging, such as the use of multiparametric magnetic resonance imaging (mpMRI) together with the Vesical Imaging-Reporting and Data System (VI-RADS), promise improvements in diagnostic accuracy. Panebianco et al. demonstrated that mpMRI achieves high diagnostic performance (pooled area under the curve of 0.90) in differentiating muscle-invasive bladder cancer (MIBC) from NMIBC (17). However, despite its potential, mpMRI is not yet a standard part of the pre-TURBT workup, as larger multicenter trials are needed to validate its widespread use. Integrating such imaging modalities may eventually refine current protocols, particularly for patients with low-risk cystoscopy features.

The question of whether immediate TURBT is necessary and whether delaying might harm the patient could be addressed through the implementation of a potential Active Surveillance (AS) strategy. AS for Ta low-grade and grade 1 tumors remains a topic of ongoing debate. In 2003, Soloway et al. were the first to propose AS as an initial treatment option for patients with small, low-grade papillary bladder tumors, suggesting it as a viable alternative to immediate surgery (18). AS offers significant cost savings and reduces the morbidity associated with repeated TURBT procedures. A 2021 meta-analysis by Petrelli et al. echoed these findings, concluding that AS was a reasonable treatment option for selected patients with low-grade NMIBC (19). However, the authors emphasized the limited data available and noted the absence of stratification by risk groups, cautioning against definitive recommendations. Similarly, the EAU guidelines recognize the weak evidence supporting AS and call for further research before widespread implementation. However, an AS protocol for bladder lesions of unclear malignant potential could be a possible solution to our question.

Our study has several limitations that should be acknowledged. First, as a retrospective analysis, it is subject to inherent biases, including selection bias and limitations in the accuracy of data collection. These limitations reflect the real-world clinical setting, where heterogeneity of patient pathways, absence of standardized risk stratification, and inconsistent use of diagnostic adjuncts such as urine cytology are common, and where documentation from both hospital and outpatient sources may vary in quality and completeness. The relatively small sample size may limit the generalizability of our findings to broader populations. Additionally, we were unable to include long-term follow-up data, which would have provided valuable insights into recurrence and progression rates beyond the first year post-TURBT. Future studies with larger sample sizes and prospective designs are needed to validate these findings and assess long-term outcomes.

## Conclusion

Direct TURBT may not be obligatory for all patients with BC suspicion. Our findings highlight the importance of thorough preoperative diagnostic tools in identifying malignancy. Plausible approaches to improve the indication setting for TURBT include accurate cystoscopic tumor evaluation, the use of more refined diagnostic techniques (cytology, PDD or NBI) and risk stratification tools to reduce reduce unnecessary procedures in low-risk patients. Additionally, second look cystoscopy should be encouraged, potentially supported by seeking second opinions to ensure a more informed decision for surgery. Further studies need to establish more selective criteria for TURBT involving larger, well-defined cohorts would be highly valuable in this context.

## Data Availability

The raw data supporting the conclusions of this article will be made available by the authors, without undue reservation.
